# Intérêt de la déxaméthasone en intrapéritonéale dans l’analgésie post cholécystectomie laparoscopique: étude prospective contrôlée randomisée en double aveugle

**DOI:** 10.11604/pamj.2023.45.14.36438

**Published:** 2023-05-04

**Authors:** Ahmed Abdelhedi, Salma Ketata, Nizar Kardoun, Mariem Keskes, Imen Zouche, Amal Ayedi, Oussema Doukeli, Mariem Khrouf, Sami Fendri, Amine Zouari, Hichem Cheikhrouhou

**Affiliations:** 1 Service d'Anesthésie Réanimation Chirurgicale, Centre Hospitalier Universitaire Habib Bourguiba Sfax, Sfax, Tunisie,; 2 Service de Chirurgie Viscérale, Centre Hospitalier Universitaire Habib Bourguiba Sfax, Sfax, Tunisie

**Keywords:** Dexaméthasone, intrapéritonéale, cholécystectomie laparoscopique, analgésie postopératoire, Dexamethasone, intraperitoneal, laparoscopic cholecystectomy, postoperative analgesia

## Abstract

**Introduction:**

l´effet des corticoïdes administrés en intraveineux sur la douleur post opératoire a été bien démontré cependant peu de travaux se sont intéressés à l´intérêt de leur utilisation en intrapéritonéal après chirurgie laparoscopique. Le but de notre travail était d´évaluer l´effet de l´instillation intra péritonéale de dexaméthasone sur l´analgésie post opératoire après cholécystectomie laparoscopique.

**Méthodes:**

nous avons mené une étude prospective randomisée contrôlée en double aveugle, incluant des patients proposés pour cholécystectomie laparoscopique programmée et randomisés en deux groupes: groupe D (ayant reçus 16 ml: 12 ml de sérum physiologique et 4 ml de solution contenant 16 mg de dexaméthasone) et groupe T (ayant reçus 16 ml de sérum physiologique). Le critère de jugement principal était: l´échelle visuelle analogique (EVA) de la douleur abdominale durant les 24 premières heures postopératoires. Les critères de jugement secondaires étaient l´incidence de la douleur de l´épaule, le délai de la première demande analgésique, la consommation de morphiniques en salle de surveillance post interventionnelle (SSPI), la consommation d´analgésiques non morphiniques et l´incidence des nausées et des vomissements durant les premières 24 heures postopératoires et la présence de complications.

**Résultats:**

soixante patients ont été inclus et répartis en deux groupes de 30. Les paramètres démographiques, la durée de l´acte opératoire et de l´anesthésie ainsi que la consommation de fentanyl en per opératoire ont été comparables entre les deux groupes. Les valeurs de l´EVA de la douleur abdominale (p≤0,001), l´incidence des douleurs au niveau de l´épaule (p<0,001), la consommation d´opioïdes et d´analgésiques (p<0,001) et l´incidence des nausées (p=0,002) et des vomissements (p=0,012) durant les premières 24 heures postopératoires ont été significativement plus basses pour le groupe D. Aucune complication liée à l´administration de dexaméthasone n´a été notée.

**Conclusion:**

la dexaméthasone en intra péritonéale a diminué les douleurs postopératoires après une cholécystectomie laparoscopique.

## Abstract (en)

**Introduction:**

the effect of intravenous corticosteroids on postoperative pain has been well demonstrated; however, few studies have focused on the efficacy of intraperitoneal corticosteroid use after laparoscopic surgery. The purpose of this study was to evaluate the effect of intraperitoneal administration of dexamethasone on postoperative analgesia after laparoscopic cholecystectomy.

**Methods:**

we conducted a prospective, randomised, double-blind, controlled study, including patients scheduled for laparoscopic cholecystectomy and randomised into two groups: the group D (receiving 16 ml: 12 ml saline and 4 ml solution containing 16mg dexamethasone) and the group T (receiving 16 ml saline). The primary endpoint was: Visual Analogue Scale (VAS) for abdominal pain during the first 24 hours after surgery. The secondary endpoints were the incidence of shoulder pain, time to first request for analgesia, the consumption of morphine in the post-intervention surveillance room (PACU), the consumption of non-opioid analgesics and the incidence of nausea and vomiting during the first 24 hours after surgery as well as the presence of complications.

**Results:**

sixty patients were included in the study and divided into two groups of 30. Demographic parameters, duration of surgical and anesthesia procedures, as well as intraoperative fentanyl consumption were comparable between the two groups. Abdominal pain VAS values (p≤0.001), the incidence of shoulder pain (p<0.001), opioid and analgesic consumption (p<0.001) and the incidence of nausea (p=0.002) and vomiting (p=0.012) during the first 24 hours after surgery were significantly lower in group D. No complications related to dexamethasone administration were noted.

**Conclusion:**

intraperitoneal dexamethasone reduces postoperative pain after laparoscopic cholecystectomy.

## Introduction

La cholécystectomie coelioscopique est actuellement la technique de référence pour le traitement de la lithiase vésiculaire simple [1]. En effet, cette approche laparoscopique offre plusieurs avantages par rapport à la laparotomie. Elle permet entre autres de minimiser le retentissement respiratoire de l´incision chirurgicale, de diminuer l´incidence des problèmes intestinaux et de réduire l´intensité et la durée de la douleur postopératoire. Cette dernière reste l´une des principales plaintes postopératoires [1]. De nombreuses études se sont intéressées à décrire la stratégie analgésique la plus adaptée à la prise en charge de la douleur postopératoire après une cholécystectomie coelioscopique. Plusieurs thérapeutiques ont été utilisées dans le cadre d´une analgésie multimodale: les anti-inflammatoires non stéroïdiens, les instillations intra péritonéales [2] ou les infiltrations sous cutanées d´anesthésiques locaux [3]. Les glucocorticoïdes sont connus pour avoir des effets anti inflammatoires, immunomodulateurs et antiémétiques. La physiologie de ces effets demeure sujette à plusieurs discussions. Plusieurs travaux portant aussi bien sur des chirurgies majeures que mineures ont montré le bénéfice de l´administration intraveineuse préopératoire de glucocorticoïdes en terme de récupération post opératoire [4]. L´effet des glucocorticoïdes en prévention des nausées et vomissements post opératoire a été clairement établi; les recommandations de Apfel [5] rendent son utilisation habituelle dans cet objectif, cependant leurs effets sur la douleur restent sujets à discussions. En effet, les études ne sont pas toutes concordantes bien que plusieurs études ont conclu à un effet bénéfique de l´injection de corticoids [4]. L´objectif principal de notre étude était d´étudier l´effet analgésique de l´injection en intra péritonéal de 16 mg de dexaméthasone après cholécystectomie laparoscopique.

## Méthodes

**Conception et cadre de l´étude:** il s´agit d´un essai clinique prospectif contrôlé randomisé, en double aveugle, portant sur des patients opérés pour cholécystectomie laparoscopique programmée suite à une lithiase vésiculaire simple.

**Population étudiée:** nous avons inclus les patients âgés entre 18 et 75 ans classés selon le score de l´American Society of Anesthesiologist (ASA) I et II, et proposés pour une cholécystectomie coelioscopique programmée de durée inférieure à 2 heures. Les critères de non inclusion étaient: un indice de masse corporelle IMC > 40 kg/m^2^, les femmes enceintes ou allaitantes, la présence d´allergie, de contre-indication à l´un des produits utilisés lors du protocole de l´étude ou des maladies gastro-intestinales. Les critères d´exclusion étaient la conversion en laparotomie, la survenue d´une complication anesthésique ou chirurgicale ou la modification du protocole d´anesthésie. Calcul de la taille de l´échantillon: la détermination du nombre de patients nécessaires a été réalisée à partir des résultats d´une pré-enquête effectuée sur 20 patients. Une diminution de l´échelle visuelle analogique (EVA) après 24 heures de l´acte opératoire de 10 mm a été considérée comme significative. En choisissant une puissance du test en bilatéral de 90% et un risque alpha de 5%, le nombre de patients nécessaires était de 26 par groupe. Nous avons alors désigné 65 patients pour avoir le nombre suffisant après d´éventuelles exclusions.

**Randomisation:** elle a été effectuée à l´entrée du bloc opératoire après vérification des critères d´inclusion et de non inclusion. Elle a été basée sur des codes générés par ordinateur conservés dans des enveloppes opaques numérotées séquentiellement selon un ratio de 1: 1. Le tirage au sort a été effectué par un médecin anesthésiste autre que celui qui a assuré l´anesthésie des patients et le suivi post opératoire. Les patients ont été répartis au hasard en 2 groupes; **groupe D:** dexaméthasone intra péritonéal: une solution de 16 ml contenant 16 mg de dexaméthasone dilués dans 12 ml de sérum physiologique (1mg/ml) a été administrée en intra péritonéal avant l´exsufflation du pneumopéritoine; pas de dexaméthasone en intra-veineux. **Groupe T:** témoin: une solution de 16 ml de sérum physiologique a été administrée en intra péritonéal avant l´exsufflation du pneumopéritoine; pas de dexaméthasone en Intra-veineux.

**Intervention:** l´étude a été menée par 3 médecins: 2 médecins anesthésistes réanimateurs, l´un a été chargé par la préparation des solutions dans les règles de l´asepsie rigoureuse selon la randomisation, tandis que l´autre a été chargé par le suivi post-opératoire et un chirurgien qui a administré la solution en intra péritonéal. A la salle opératoire, tous les patients étaient monitorés par un électrocardioscope, une pression artérielle non invasive et un saturomètre et PetCO_2_. Une voie veineuse 20 gauge a été mise en place et un pré remplissage par 500CC de sérum physiologique a été débuté. Après dénitrogénation au masque à 100% d´oxygène durant 3 minutes, une anesthésie générale a été induite avec 3 µg/kg de fentanyl, 3 mg/kg de propofol et 0,15mg/kg de cisatracurium suivie d´une ventilation au masque pendant 3 minutes puis intubation orotrachéale par sonde numéro 7 pour les femmes et sonde numéro 7,5 pour les hommes. L´entretien a été fait par des réinjections de fentanyl 50µg et de cisatracurium 0.03 mg/kg toutes les 30 minutes avec le sevoflurane 2% à 3% dans 50% d´oxygène/air. Le patient a été mis sous ventilation à volume assisté contrôlé. Une sonde nasogastrique a été insérée pour vider l´air de l´estomac. Au cours de la coelioscopie, la cavité péritonéale a été insufflée de dioxyde de carbone pour maintenir une pression intra-abdominale <12 mmHg. A la fin de l´acte, la seringue préparée dans les règles d´asepsie rigoureuse par le médecin qui a fait la randomisation, contenant 16 ml de dexaméthasone dilué ou du sérum physiologique, selon la randomisation, a été administrée par le chirurgien qui a été aveuglé de la composition de la seringue en intrapéritonéal et en sous diaphragmatique avant l´exsufflation du pneumopéritoine. Trente minutes avant l´extubation, 1 g de paracétamol (perfalgan), avec 20 mg de nefopam pendant 2 heures puis au service de chirurgie. A cette phase, un autre médecin anesthésiste autre que celui qui a fait la randomisation et la préparation de la seringue a été chargé du recueil des données post opératoires. A la SSPI, une surveillance de la fréquence cardiaque, de la saturation pulsée d´oxygène, et de la pression artérielle a été effectuée toutes les 15 minutes pendant 2 heures.

L´échelle EVA a été utilisée pour l´évaluation des douleurs postopératoires. Elle a été expliquée aux patients dès l´inclusion au protocole. Elle consiste en une règle de 10 cm de longueur dont les extrémités représentent d´un côté l´absence de douleur (0) et de l´autre la douleur maximale inimaginable et intolérable (10). Le patient côte l´intensité de sa douleur en déplaçant un curseur entre les deux extrémités. Une graduation au verso de la réglette permet d´évaluer le score de la douleur. La présence de douleurs avec EVA >30 nécessite une titration de morphine par une dose de charge de 3 mg en intraveineux direct puis des injections répétées de 2 mg toutes les cinq minutes jusqu´à l´obtention d´une EVA<30. Au service de chirurgie viscérale, un suivi a été effectué toutes les 6 heures pendant les 24 premières heures post opératoires. Tous les patients ont reçu 1g de perfalgan toutes les 6 heures en post opératoire. La présence de douleurs avec EVA >30 nécessite l´administration 20 mg de nefopam (Acupan) et si une elle persiste une dose de 50mg de tramadis en sous-cutané a été administrée. Tous les patients ont été invités à signaler les éventuels effets indésirables du dexamethasone tels que des vertiges, des maux de tête, des douleurs au cou, aux épaules ou des évanouissements pendant les 24 premières heures postopératoires, ou des complications retardées, telles qu´une infection de la plaie opératoire ou un retard de cicatrisation au cours de la première semaine postopératoire.

**Les données recueillies:** en préopératoire, nous avons recueilli l´âge, le poids, la taille, l´indice de masse corporelle (IMC), la classe ASA, et les antécédents médicaux et chirurgicaux. En peropératoire, nous avons recueilli la durée de l´acte qui a été calculée à partir du moment de l´incision ombilicale pour l´insertion du trocart jusqu´au dernier point de suture, la durée de l´anesthésie qui a été calculée à partir de l´induction jusqu´à l´extubation, les paramètres hémodynamiques et la dose totale de fentanyl en peropératoire. En post opératoire, nous avons recueilli les paramètres hémodynamiques toutes les 15 minutes pendant les 2 premières heures. Nous avons recueilli aussi L´EVA de la douleur abdominale, l´incidence de la douleur de l´épaule, l´incidence des nausées et des vomissements (NVPO) et les doses totales des antiémétiques de secours à H2, H6, H12, H18 et H24 postopératoire. A la fin nous avons recueilli les effets indésirables de la dexaméthasone, les éventuelles complications post opératoires, le délai de la première prise d´antalgique, la dose totale des antalgiques, la dose totale de morphine, le délai du premier lever et le délai de reprise de transit.

**Critères de jugement:** notre critère de jugement principal était l´EVA de douleurs au site opératoire durant les premières 24 heures postopératoires. Les critères de jugement secondaires étaient l´incidence des douleurs au niveau de l´épaule, la consommation d´antalgiques en post-opératoire, le délai de la première demande d´analgésique, l´incidence des nausées et des vomissements durant les premières 24 heures postopératoires, et la réhabilitation post opératoire.

**Etude statistique:** la saisie et l´analyse des données ont été réalisées avec SPSS version 25.0. L´étude descriptive des variables qualitatives a été exprimée en pourcentages (%). Pour les variables quantitatives, l´étude de la distribution des données a été faite par le test de Shapiro-Wilk. La description de ces variables a été faite par les moyennes et écart type en cas de distribution normale et par les médianes et la plage interquartile dans le cas contraire. Pour l´analyse comparative, nous avons utilisé le test de chi-2 de Pearson pour la comparaison de deux fréquences en cas de conditions d´application vérifiées et le test de chi-2 de Fischer dans le cas contraire. Pour l´analyse des variables quantitatives, nous avons utilisé le test de Student pour la comparaison de deux moyennes en cas de distribution normale et le test non paramétrique de Mann-Witney dans le cas contraire. Dans toutes les comparaisons, la valeur de p ≤ 0,05 a été considérée comme statistiquement significative.

**Considération éthique:** cet essai clinique a été réalisé après accord du comité de protection des personnes sud (C.P.P.SUD) sous l´égide des ministères de la santé et de la justice de la république tunisienne qui s´est réuni à la date du lundi 17 juin 2020 (référence CPP SUD N° 0242/2020) et après consentement éclairé et écrit des patients inclus.

## Résultats

**Figure 1:**
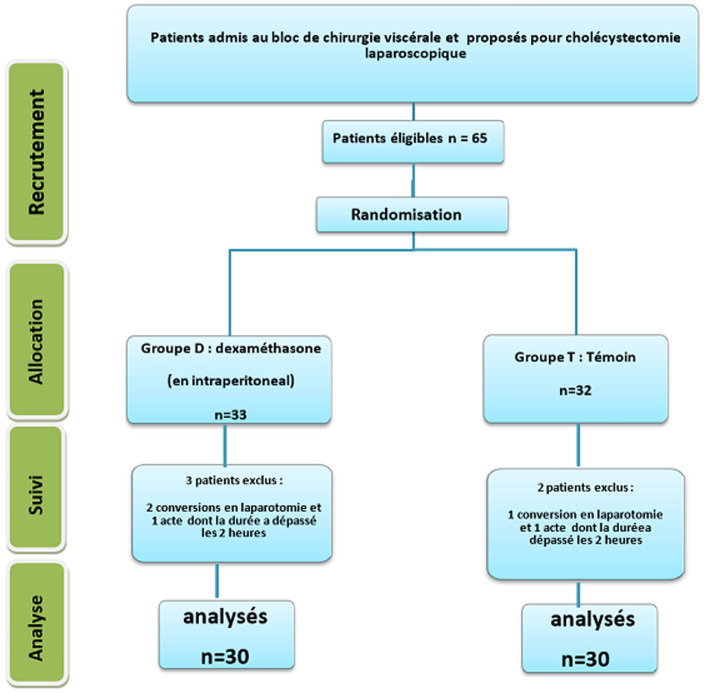
diagramme de flux

**Caractéristiques générales de la population étudiée:** nous avons inclus 65 patients dont 5 patients ont été exclus: trois (3) pour conversion en laparotomie et 2 pour une durée d´acte supérieure à 2 heures. Soixante (60) patients ont été analysés et répartis en deux groupes de 30 (Figure 1). La population étudiée présentait une médiane d´âge de 48,9 ans [22,74], un sexe ratio à 0,5 et un IMC moyen de 26,86±2,9 kg/m^2^. Aucune différence significative des paramètres démographiques et comorbidités n´a été observée entre les deux groupes de notre étude (Tableau 1). La durée de l´acte opératoire et de l´anesthésie ainsi que la consommation de fentanyl en per opératoire ont été comparables entre les 2 groupes (Tableau 1). La pression d´insufflation n´a pas dépassé 12 mmHg dans les deux groupes de l´étude sans différence significative entre les deux groupes.

**Tableau 1:** comparaison des paramètres pré et peropératoires entre les 2 groupes

	Groupe D (n=30)	Groupe T (n=30)	P
**Age (ans)±ET**	50,97±15,96	46,83±10,78	0,245**
**Sexe (H/F)**	13/17	7/23	0,100*
**IMC(Kg/m^2^)±ET**	26,61±3,85	27,11±2,54	0,554**
**ASA (I/II)**	19/11	21/9	0,584*
**HTA**	7	7	1,000*
**Diabète**	6	2	0,254*
**Durée moyenne de l’acte chirurgical (minutes) ± ET**	93±24,58	92,17±19,5	0,885**
**Durée moyenne de l’anesthésie (minutes) ± ET**	106.40±26	101,09±21	0,76**
**Dose moyenne de Fentanyl (µg/kg) ± ET**	4,28±0,809	3,97±0,742	0,123**

Groupe D: dexaméthasone intra péritonéal; Groupe T: placebo; n: nombre; H: Homme, F: Femme; IMC: indice de masse corporelle; ASA: American Society of Anesthesiologists; HTA : hypertension artérielle, ET: Ecart Type; Tests statistiques: **: Test de Student; *:Test de chi-2 de Pearson

**Analgésie et réhabilitation post opératoire:** un postopératoire, une diminution hautement significative de l´EVA à la douleur abdominale a été observée chez le groupe D par rapport au groupe T à H2, H6, H12 et H24 (p < 0,001) (Tableau 2). Par ailleurs, dans le groupe D, il y avait une diminution significative de l´incidence de la douleur de l´épaule (13,3%) par rapport au groupe T (70%) avec p<0,001, une réduction significative de la consommation d´antalgiques et de la morphine en post opératoire (P<0,001) avec un allongement significatif du délai de la 1^ère^ demande d´antalgique par rapport au groupe T (P<0,001) (Tableau 3). Les nausées (p=0,002), les vomissements (p=0,012) et l´administration d´antiémétiques (P=0,007) en post opératoire ont été significativement diminués chez le groupe D par rapport au groupe T (Tableau 3). Une diminution significative du délai du premier lever (p=0,001) et de la reprise du transit (p=0,038) a été observée en faveur du groupe D par rapport au groupe T (Tableau 3).

**Tableau 2:** comparaison de l’EVA de la douleur abdominale postopératoire entre les 2 groupes

	Groupe D (n=30)	Groupe T (n=30)	p
**EVA(H2)± ET**	26,3±10,98	58,7±19,78	< 0,001
**EVA(H6)±ET**	24,3±8,17	52±19,19	< 0,001
**EVA(H12)± ET**	19,7±7,65	36,7±17,68	< 0,001
**EVA(H24)± ET**	13,7±8,9	24,7±15,25	0,001

Groupe D: dexaméthasone intra péritonéal; Groupe T: Témoin; n=nombre; EVA: échelle visuelle analogique, ET: Ecart Type Tests statistiques

**Tableau 3:** comparaison des paramètres postopératoires entre les 2 groupes

		Groupe D (n=30)	Groupe T (n=30)	p
**Douleur de l’épaule**		4 (13,3%)	21 (70%)	< 0,001*
**Délai de la 1^ère^ demande d’analgésiques (min)[]**		212,5 [20-420]	56,17 [10-360]	< 0,001†
**Recours à la Morphine**		11 (36,7%)	28(93,3%)	< 0,001*
**Recours au Nefopam**		7(36,7%)	21 (70%)	< 0,001*
**Recours au TRAMAL^®^**		0(0%)	10(33,3%)	0,001*
**Dose moyenne de morphine administrée (mg)±ET**	**H1**	2,73±0,905	3,5±1,139	0,036*
	**H2**	0,55±0,934	1,46±1,105	0,016*
**Nausées**		9(30%)	21(70%)	0,002*
**Vomissement**		5 (16,7%)	14(56,7%)	0,012*
**Prise d’antiémétiques**		6(20%)	16(53,3%)	0,007*
**Délai du 1^er^ lever (H)±ET**		14,27±5,626	18,87±4,125	0,001**
**Délai de reprise du transit (H)±ET**		12,53±6,627	16±6,023	0,038**

Groupe D: dexaméthasone intra péritonéal ; Groupe T : Témoin ; ET: Ecart Type; [ ]: extrêmes , H: heure tests statistiques: *: Test de Chi-2 de Pearson; †: Test de Mann-Whitney ; **: Test de Student.

**Effets indésirables:** les paramètres hémodynamiques per et postopératoires ont été comparables entre les 2 groupes. Aucune complication métabolique ou infectieuse liée à l´administration intra péritonéale de dexaméthasone n´a été enregistrée.

## Discussion

Nous avons mené un essai clinique prospectif randomisé en double aveugle dont l´objectif était d´étudier l´effet analgésique de l´injection en intra péritonéal de 16 mg de dexaméthasone après cholécystectomie laparoscopique. Pour ce faire nous avons inclus 60 patients proposés pour cholécystectomie laparoscopique et randomisés en deux groupes de 30: Groupe D (Dexaméthasone intrapéritonéal) et Groupe T (Témoin). Notre étude a montré que la dexaméthasone en intrapéritonéale a amélioré l´analgésie post opératoire en diminuant les douleurs abdominales, scapulaires, et la consommation de morphine et d´analgésiques tout en allongeant le délai de la 1^ère^ prise d´antalgique en post opératoire. Chez 26 à 41% des patients après une cholécystectomie coelioscopique, la douleur est la principale raison de la prolongation du séjour à l´hôpital et est la cause majeure d´une convalescence prolongée [6]. La douleur postopératoire secondaire à une cholécystectomie coelioscopique dépend de plusieurs facteurs. On peut en citer la rupture de vaisseaux sanguins suite à la distension rapide du péritoine, la traction nerveuse traumatique, la libération de molécules inflammatoires, le traumatisme de la paroi abdominale provoqué par l´insertion des trocarts, le traumatisme survenant lors du retrait de la vésicule biliaire de l´abdomen, le pneumopéritoine créé par l´utilisation de CO_2_, le maintien d´une pression abdominale élevée, l´irritation du nerf phrénique et l´application de CO_2_ froid [7] .

Etant donné que les glucocorticoids (GC) pourraient agir sur plusieurs composantes de la réponse inflammatoire à la chirurgie, l´attention s´est portée sur la capacité des GC à moduler ces réponses potentiellement délétères, et donc la possibilité d´atténuer la douleur post opératoire [8]. En effet, les effets anti-inflammatoires périphériques des glucocorticoïdes résultent de l´inhibition de l´action des phospholipases, en bloquant la synthèse de l´acide arachidonique et par conséquent la production de prostaglandines et de leucotriènes. Les corticoïdes inhibent également l´action des COX-2 [6]. Les corticostéroïdes bloquent aussi la production de cytokines pro-inflammatoires comme l´interleukine-1, l´interleukine-6 et le TNFÞ ainsi que celle de bradykinine et stabilisent rapidement les membranes neuronales ce qui peut exercer un effet favorable sur les mécanismes neurogéniques de l´inflammation postopératoire. Des études expérimentales ont montré que les effets des glucocorticoïdes sont rapides et obtenus en moins de 15 minutes [9,10]. Karanicolas *et al*. [11] a publié une méta-analyse de 17 essais contrôlés randomisés, et a évalué l´impact de l´administration prophylactique de la dexaméthasone en intraveineux (IV) sur les symptômes postopératoires chez les patients subissant une cholécystectomie laparoscopique. Les auteurs ont conclu que la dexaméthasone intraveineuse a réduit considérablement l´incidence de la douleur postopératoire et des nausées et des vomissements. Dans une étude incluant 88 patients proposés pour une cholécystectomie coelioscopique, Bisgaard *et al*. [12] ont trouvé que l´injection de 8 mg de dexaméthasone en intraveineux 90 minutes avant l´induction a diminué significativement des scores de la douleur abdominale postopératoire à H6, H12 et H24. Par contre, ils n´ont pas signalé une diminution statistiquement significative des chiffres de l´EVA à H0, H1, H2 et H3 postopératoire. Par ailleurs, dans une étude randomisée portant sur 70 patients proposés pour cholécystectomie laparoscopique évaluant l´effet de l´injection intraveineuse de 8 mg de dexaméthasone, Sistla [9] a montré que le score médian de la douleur au repos dans le groupe recevant la dexaméthasone a été significativement plus faible que celui du groupe placebo à H 24 (EVA 2 contre EVA 4, P=0,002). De plus, le score médian de la douleur à l´effort a été également moins élevé dans le groupe recevant la dexaméthasone avec une différence significative (EVA 3 contre EVA 4,5, P=0,001).

Jusqu´à ce jour, peu d´études ont comparé l´effet de la dexaméthasone par voie intra-péritonéale sur les douleurs abdominales post opératoires. La majorité s´est intéressée à la voie IV. Le seul travail utilisant la dexaméthasone par voie intra-péritonéale est celui publié par Asgari *et al*. [13] portant sur des patientes programmées pour une chirurgie gynécologique par voie cœlioscopique. Les patientes étaient réparties en deux groupes: le groupe intra-péritonéal ayant reçu 16 mg de dexaméthasone par voie intra péritonéale et le groupe placebo ayant reçu 16 ml de sérum physiologique par voie intra péritonéale. Les auteurs ont conclu que l´intensité de la douleur a été significativement plus faible dans le groupe dexaméthasone, pendant le séjour en SSPI et 2, 4, 8, 12 et 24 heures après la chirurgie, que dans le groupe placebo. En plus, ils ont objectivé que la quantité d´opioïde prescrite comme analgésique dans le groupe placebo (35,9 ± 18,9) était significativement plus importante que celle utilisée dans le groupe dexaméthasone (27,5 ± 7,6 mg) (p=0,025). Dans une méta analyse récente incluant 32 études (3284 femmes), Kaloo *et al*. [10] a étudié les interventions permettant de diminuer la douleur scapulaire post chirurgie coelioscopique gynécologique et a conclu que l´instillation de fluide (cristalloïde) dans la cavité péritonéale a réduit l´incidence et la sévérité des douleurs scapulaires à 24 heures post opératoires. Par ailleurs, les auteurs ont trouvé que l´instillation d´un anesthésique locale dans la cavité péritonéale a réduit l´incidence des douleurs scapulaires 4 à 8 heures post opératoire; par contre, l´anesthésie locale sous-diaphragmatique a probablement peu ou pas d´effet sur la douleur de l´épaule.

Notre étude a montré que la dexaméthasone intrapéritonéale a réduit les nausées et les vomissements post opératoires (NVPO), le délai du premier lever et de la reprise du transit contribuant ainsi à une meilleure réhabilitation post opératoire. En effet, la dexaméthasone, seule ou en association avec d´autres médicaments antiémétiques a également été rapportée efficace pour la prévention des nausées et des vomissements après une cholécystectomie laparoscopique [14,15]. Dans l´essai de Fukami *et al*. [6] évaluant l´effet de l´injection en IV de 8 mg de dexaméthasone chez les patients subissant une cholécystectomie laparoscopique, l´incidence des NVPO dans le groupe placebo était de 40%, contre 18% dans le groupe dexaméthasone avec une différence statistiquement significative. Il n´existe pas d´étude qui évalue l´effet de la dexaméthasone en intrapéritonéale sur les NVPO. Selon Murphy [1,6], parmi les patients subissant une cholécystectomie laparoscopique en ambulatoire, l´utilisation de 8 mg de dexaméthasone préopératoire en intraveineux a amélioré la qualité du rétablissement après la sortie de l´hôpital, et a réduit les nausées, la douleur et la fatigue dans la période postopératoire précoce. Notre étude a mis en exergue une technique analgésique simple à réaliser, nouvelle, efficace et dénuée de complications post opératoires ouvrant ainsi de nouvelles perspectives en ce qui concerne la cholécystectomie comme étant une chirurgie ambulatoire. Cependant, cette étude n´est pas dénudée de limites étant donné qu´il s´agit d´un travail mono centrique avec des opérateurs différents.

## Conclusion

Notre étude suggère que la dexaméthasone en intra péritonéale est une technique efficace pour diminuer les douleurs et améliorer la réhabilitation post opératoire après une cholécystectomie laparoscopique.

### Etat des connaissances sur le sujet

- *Après une cholécystectomie coelioscopique, la douleur est la principale raison de la prolongation du séjour à l´hôpital;*
- *La dexaméthasone en intraveineux réduit les la douleur postopératoire chez les patients subissant une cholécystectomie laparoscopique;*
- *La dexaméthasone en intraveineux réduit l´incidence des nausées et des vomissements post opératoires*.

### Contribution de notre étude à la connaissance

- *La dexaméthasone en intrapéritonéale réduit la douleur postopératoire chez les patients subissant une cholécystectomie laparoscopique;*
- *La dexaméthasone en intrapéritonéale réduit l´incidence des nausées et des vomissements post opératoires chez les patients subissant une cholécystectomie laparoscopique;*
- *La dexaméthasone en intrapéritonéale chez les patients subissant une cholécystectomie laparoscopique diminue le délai du premier lever et le délai de la reprise du transit ce qui favorise une meilleure réhabilitation post opératoire*.
